# Dim light at night disrupts the sleep-wake cycle and exacerbates abnormal EEG activity in *Cntnap2* knockout mice: implications for autism spectrum disorders

**DOI:** 10.1186/s13229-025-00689-7

**Published:** 2025-12-18

**Authors:** Yumeng Wang, Ketema N. Paul, Gene D. Block, Tom Deboer, Christopher S. Colwell

**Affiliations:** 1 Department of Psychiatry & Biobehavioral Sciences, University of California Los Angeles, Los Angeles, CA USA; 2 Department of Integrative Biology and Physiology, University of California Los Angeles, Los Angeles, CA USA; 3 Laboratory for Neurophysiology, Department of Cell and Chemical Biology, Leiden University Medical Center, Leiden, The Netherlands

**Keywords:** Autism spectrum disorder, Dim light at night, Epilepsy, Seizure, Mice, Sleep-wake rhythm, Sex difference

## Abstract

**Background:**

Epilepsy is a common comorbidity in individuals with autism spectrum disorders (ASDs). Many patients with epilepsy as well as ASD experience disruptions in their sleep-wake cycle and daily fluctuations in symptom severity. Chronic exposure to light at nighttime can disrupt sleep and circadian rhythms. Contactin associated protein-like 2 knockout (*Cntnap2* KO) mice, a model of ASD and epilepsy, exhibit sleep and circadian disturbances and abnormal events in the electroencephalogram (EEG). Here, we investigated how chronic dim light at night (DLaN) exposure affects sleep architecture, EEG power spectra, and abnormal EEG events in *Cntnap2* KO and wildtype (WT) mice.

**Methods:**

Male and female *Cntnap2* KO and WT mice were exposed to DLaN (5 lx) for 6 weeks. EEG recordings were collected and analyzed to assess sleep architecture, spectral power, and abnormal EEG events. A two-way repeated-measures analysis of variance (ANOVA) was used to evaluate the effects of DLaN across time and EEG frequencies, followed by Bonferroni-corrected post hoc tests where appropriate.

**Results:**

DLaN exposure delayed wake onset and disrupted sleep patterns in a sex-dependent manner, with females being more affected. DLaN significantly increased slow-wave activity (SWA, 0.5–4 Hz) in both WT and KO mice, consistent with increased sleep pressure. Notably, DLaN markedly elevated the frequency of abnormal hypersynchronized EEG events in *Cntnap2* KO mice and even induced such events in WT mice. Spectral analysis of abnormal EEG events revealed elevated theta power, suggesting hippocampal involvement.

**Conclusions:**

Chronic DLaN exposure disrupts sleep architecture in a sex-dependent fashion and increases the occurrence of abnormal EEG events in *Cntnap2* KO mice. These findings highlight the potential risks of nighttime light exposure for individuals with ASD and epilepsy, underscoring the importance of managing light environments to improve sleep quality and neurological health.

**Supplementary Information:**

The online version contains supplementary material available at 10.1186/s13229-025-00689-7.

## Introduction

Epilepsy is a common comorbidities in individuals with autism spectrum disorder (ASD) [1, 2]. A systematic review and meta-analysis evaluating 66 studies, ranging from clinic samples to population samples, reported a pooled prevalence rate of epilepsy in 7% of children and 19% of adults with ASD [3]. Individuals with ASD and epilepsy tend to exhibit higher rates of intellectual disability and broader neurodevelopmental and psychiatric impairments compared with individuals with ASD without epilepsy [4–7]. Other studies have reported higher rates of epileptiform discharges and other abnormal electroencephalographic (EEG) features in individuals with ASD, even without the presence of clinical seizures. Interestingly, studies using overnight EEG monitoring in children with ASD reported higher rates of interictal epileptiform discharges during sleep [8–11]. These findings fit into a larger literature indicating that many patients with epilepsy experience disruptions in their sleep-wake cycle and exhibit daily rhythms in symptom expression [12–14]. Sleep disturbances are commonly reported in ASD, with affected individuals experiencing delayed sleep onset, nighttime arousals, fragmented sleep, and reduced total sleep duration [15–17]. The co-occurrence of epilepsy further exacerbates sleep disturbances. These associations raise questions about the underlying mechanisms, such as the shared role of pathological excitability and altered synaptic transmission that are best addressed using preclinical models [18].

To examine how autism-like behaviors, epilepsy, and sleep disturbances intersect, we explored a mouse model that exhibited all three phenotypes [19, 20]. Prior work has shown that mutations in the contactin associated protein-like 2 (*Cntnap2*) gene are associated with ASD, cortical dysplasia-focal epilepsy (CDFE), intellectual disabilities, and seizures in patients [21–28]. Similarly, a *Cntnap2* KO mouse model [29] has been shown to have seizure-like activity, frequent interictal discharges, social behavioral deficits, and repetitive behaviors [30–33]. Previous studies have shown that *Cntnap2* KO mice have disrupted sleep, and dampened day-night activity rhythms compared to wildtype (WT) mice [34–36]. Chronic exposure to light at nighttime has been associated with disrupted daily activity, delayed sleep onset, a dampened melatonin profile, mood alterations, metabolic dysfunctions, and poor cognition performance [39, 41, 42, 47, 48]. We have previously shown that these mice are vulnerable to exposure to a mild yet common circadian disruption from exposure to dim light at night (DLaN). This disruption impacted both activity rhythms and reduced social interactions and increased repetitive behaviors [36]. The negative effects of DLaN were reduced by treatment with melatonin [36] or by shifting the light toward warmer colors that minimized stimulation of melanopsin [37]. However, the effects of DLaN on EEG-defined sleep and epileptiform activity have yet to be explored.

In the present study, we used EEG/ Electromyography (EMG) recordings to measure daily rhythms in vigilance states – wake, non-rapid eye movement (NREM) sleep, and rapid eye movement (REM) sleep – in male and female *Cntnap2* KO and WT mice. Following baseline recordings under standard 12:12 light/dark conditions, the mice were exposed to DLaN for six weeks. We were particularly interested in evaluating sex-dependent differences in the temporal patterns of vigilance states. In addition, we wanted to test the hypothesis that the *Cntnap2* KO mice would be vulnerable to circadian disruption induced by DLaN exposure. We also evaluated the impact of this environmental perturbation on the power spectrum of NREM sleep, particularly focusing on the slow-wave activity range (SWA, 0.5–4 Hz). Finally, we sought to determine if DLaN exposure would impact the frequency of abnormal EEG activity in the *Cntnap2* KO mice.

## Results

### Fragmented sleep and dampened daily sleep-wake rhythm in the *Cntnap2* KO mice

**Fig. 1:**
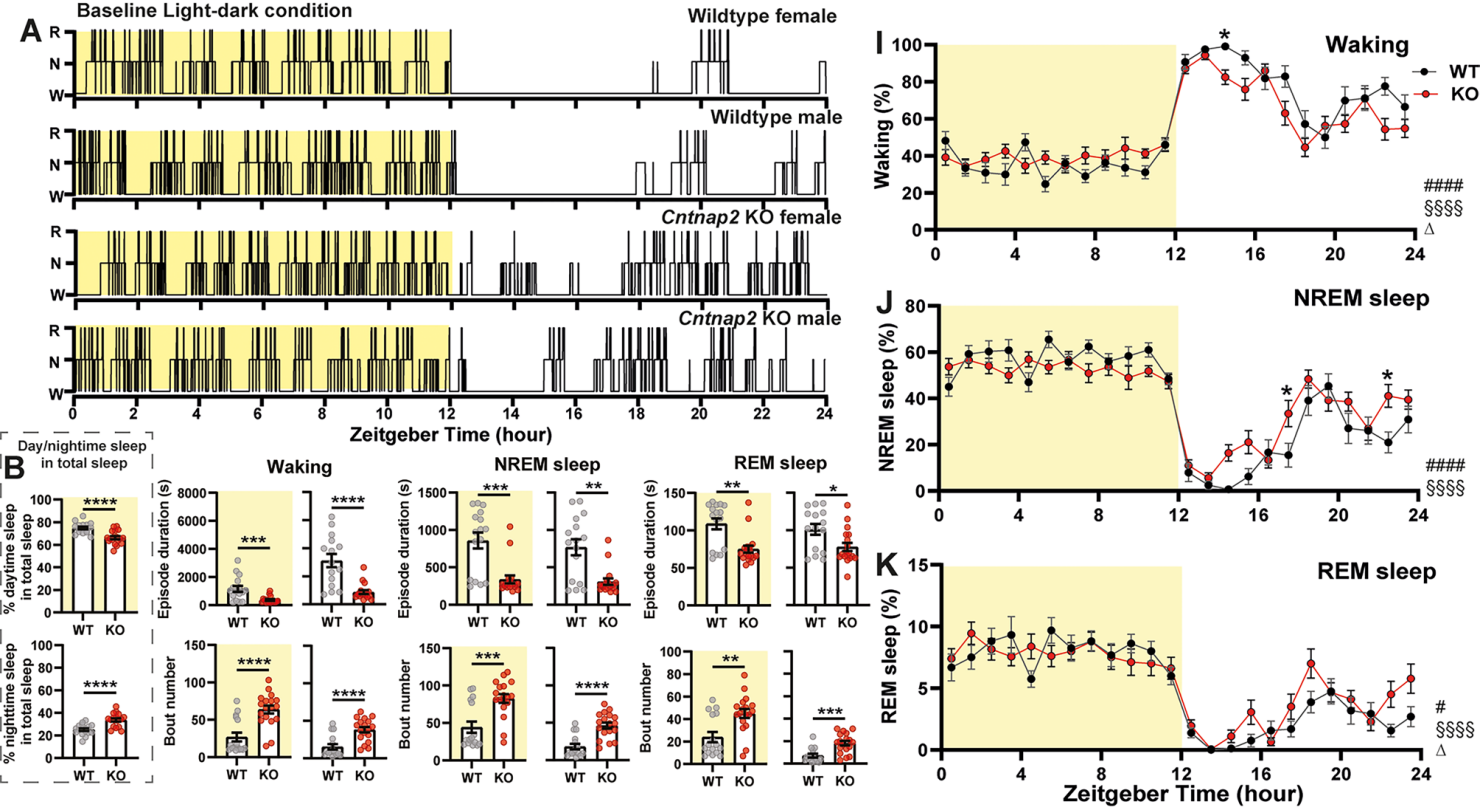
*Cntnap2* KO mice have more fragmented sleep compared with WT mice. (**A**) Representative 24-hr hypnograms. Yellow shading indicates the light phase. R: REM sleep, N: NREM sleep, W: wakefulness. (**B**) Percentage of light-phase (upper) and dark-phase (bottom) sleep relative to total sleep in WT (grey circles) and KO (red circles) mice. (**C – H**) Episode duration and bout numbers of wakefulness (**C**,** F**), NREM sleep (**D**,** G**) and REM sleep (**E**, **H**) of WT mice and KO mice during the light phase (left) and dark phase (right). Asterisks indicate significant differences between WT and KO mice (unpaired t-test or Mann-Whitney U test; *p < 0.05, **p < 0.01, ***p < 0.001, ****p < 0.0001). (**I-K**) Twenty-four-hour distribution of time spent in wakefulness (**I**), NREM sleep (**J**), and REM sleep (**K**) for WT (black) and KO (red) mice. Pound sign (#) indicates a significant interaction between the factors “zeitgeber time” and “genotype” (two-way repeated-measures ANOVA with Geisser-Greenhouse’s correction, #*p* < 0.05, ####*p* < 0.0001). Main effect of zeitgeber time and genotype were showed as “§” and “∆” (§§§§*p* < 0.0001, ∆*p* < 0.05). Asterisks in panel I-K indicate significant post-hoc differences between WT and KO mice (Bonferroni multiple comparisons test, *p < 0.05). Sample size: WT, 8 male and 8 female; KO, 10 male and 8 female. Data are shown as mean ± SEM

Prior work has found evidence for disturbed sleep/wake rhythms in male *Cntnap2* KO mice [34–36]. In the present study, we used EEG measurements to determine whether the mutation impacted daily rhythms of sleep-wake architecture in *Cntnap2* KO and WT mice. As illustrated by the hypnograms, *Cntnap2* KO mice exhibited significantly more fragmented vigilance states and increased sleep during the dark phase (Fig. 1A). We did not observe strong sex differences in either the WT or KO groups; therefore, the data are presented as mixed sex. Detailed statistical results for sex differences are provided in Table 1 and Supplemental Table 1.

**Table 1:** Vigilance state EEG measurements analyzed with three-way ANOVA data with sex, genotype and time as factors. Measurements were made with mice under LD 12:12 conditions and again after 6 weeks exposure to dim light at night (DLaN)

Measurement	Time	Genotype	Sex	Time x Genotype	Time x Sex	Genotype x Sex	Time x Genotype x Sex
Waking (%) LD	*F*_(23, 216)_ = 38.29; ***p*** **< 0.0001**	*F*_(1, 216)_ = 3.903; ***p*** **= 0.0495**	*F*_(1, 216)_ = 1.488	*F*_(23, 216)_ = 3.009; ***p*** **< 0.0001**	*F*_(23, 216)_ = 1.027	*F*_(1, 72)_ = 0.051	*F*_(23, 72)_ = 1.023
NREM sleep (%) LD	*F*_(23, 216)_ = 36.38; ***p*** **< 0.0001**	*F*_(1, 216)_ = 2.962	*F*_(1, 216)_ = 0.851	*F*_(23, 216)_ = 3.092; ***p*** **< 0.0001**	*F*_(23, 216)_ = 1.001	*F*_(1, 72)_ = 0.1469	*F*_(23, 72)_ = 1.077
REM sleep (%) LD	*F*_(23, 216)_ = 22.39; ***p*** **< 0.0001**	*F*_(1, 216)_ = 3.905; ***p*** **= 0.0494**	*F*_(1, 216)_ = 5.255; ***p*** **= 0.0228**	*F*_(23, 216)_ = 1.693; ***p*** **= 0.0288**	*F*_(23,216)_ = 0.744	*F*_(1, 72)_ = 0.227	*F*_(23, 72)_ = 1.055
Waking (%) DLaN	*F*_(23, 192)_ = 33.47; ***p*** **< 0.0001**	*F*_(1, 192)_ = 1.574	*F*_(1, 192)_ = 17.23; ***p*** **< 0.0001**	*F*_(23, 192)_ = 2.958; ***p*** **< 0.0001**	*F*_(23, 192)_ = 3.033; ***p*** **< 0.0001**	*F*_(1, 48)_ = 0.713	*F*_(23, 48)_ = 1.525
NREM sleep (%) DLaN	*F*_(23, 192)_ = 30.77; ***p*** **< 0.0001**	*F*_(1, 192)_ = 0.362	*F*_(1, 192)_ = 14.92; ***p =*** **0.0002**	*F*_(23, 192)_ = 3.155; ***p*** **< 0.0001**	*F*_(23, 192)_ = 3.034; ***p*** **< 0.0001**	*F*_(1, 48)_ = 0.118	*F*_(23, 48)_ = 1.417
REM sleep (%) DLaN	*F*_(23, 192)_ = 26.17; ***p*** **< 0.0001**	*F*_(1, 192)_ = 13.42; ***p*** **= 0.0003**	*F*_(1, 192)_ = 15.22; ***p*** **< 0.0001**	*F*_(23, 192)_ = 0.971	*F*_(23, 192)_ = 2.423; ***p*** **= 0.0006**	*F*_(1, 48)_ = 8.566; ***p*** **= 0.0052**	*F*_(23, 48)_ = 1.352

Quantification of sleep percentages across the light and dark phases confirmed these findings, with KO mice spent less time asleep during the light phase and more time asleep during the dark phase compared to WT controls (Fig. 1B). Previous studies have shown that episode duration of wake is shorter in male KO mice compared to WT, with a corresponding increase in wake bouts [34]. Further analysis revealed that, regardless of the light or dark phase, the average episode duration of wake, NREM sleep, and REM sleep was significantly shorter in KO mice than in WT mice (Fig. 1C-E); similarly the bout number for each vigilance is higher in the KO mice than in the WT mice, further supporting the presence of sleep fragmentation (Fig. 1F-H). The 24-h time courses of time spent in each state indicate a blunted daily sleep rhythm, with significant interactions between Genotype and Zeitgeber time for wakefulness (*F*_23, 736_ = 2.798, *p* < 0.0001; Fig. 1I), NREM sleep (*F*_23, 736_ = 2.889, *p* < 0.0001; Fig. 1J) and REM sleep (*F*_23, 736_ = 1.644, *p* = 0.030; Fig. 1K). Post-hoc analysis indicated that KO mice slept more during the dark phase (Fig. 1I, J). Notably, wake and REM sleep distribution over the 24-hour cycle was affected by genotype (wake: *F*_1, 32_ = 4.386, *p* = 0.044; REM sleep: *F*_1, 32_ = 4.592, *p* = 0.040). Overall, our EEG based sleep/wake data confirmed that the *Cntnap2* KO mice exhibited sleep fragmentation with a significant impact on the daily rhythms for all states and an increase in dark-phase sleep, leading to a blunted daily sleep rhythm.

### Sleep architecture is more affected by DLaN in *Cntnap2* KO mice in a sex dependent manner

To investigate the effects of DLaN on sleep architecture, we exposed mice to 5 lx dim light during the dark phase, comparable to the intensity of light emitted by electronic devices in a dark room, and 300 lx during the light phase, typical of working environments. We evaluated sleep architecture in *Cntnap2* KO and WT mice over a 24-hr period following 2 and 6 weeks of DLaN exposure.

**Fig. 2:**
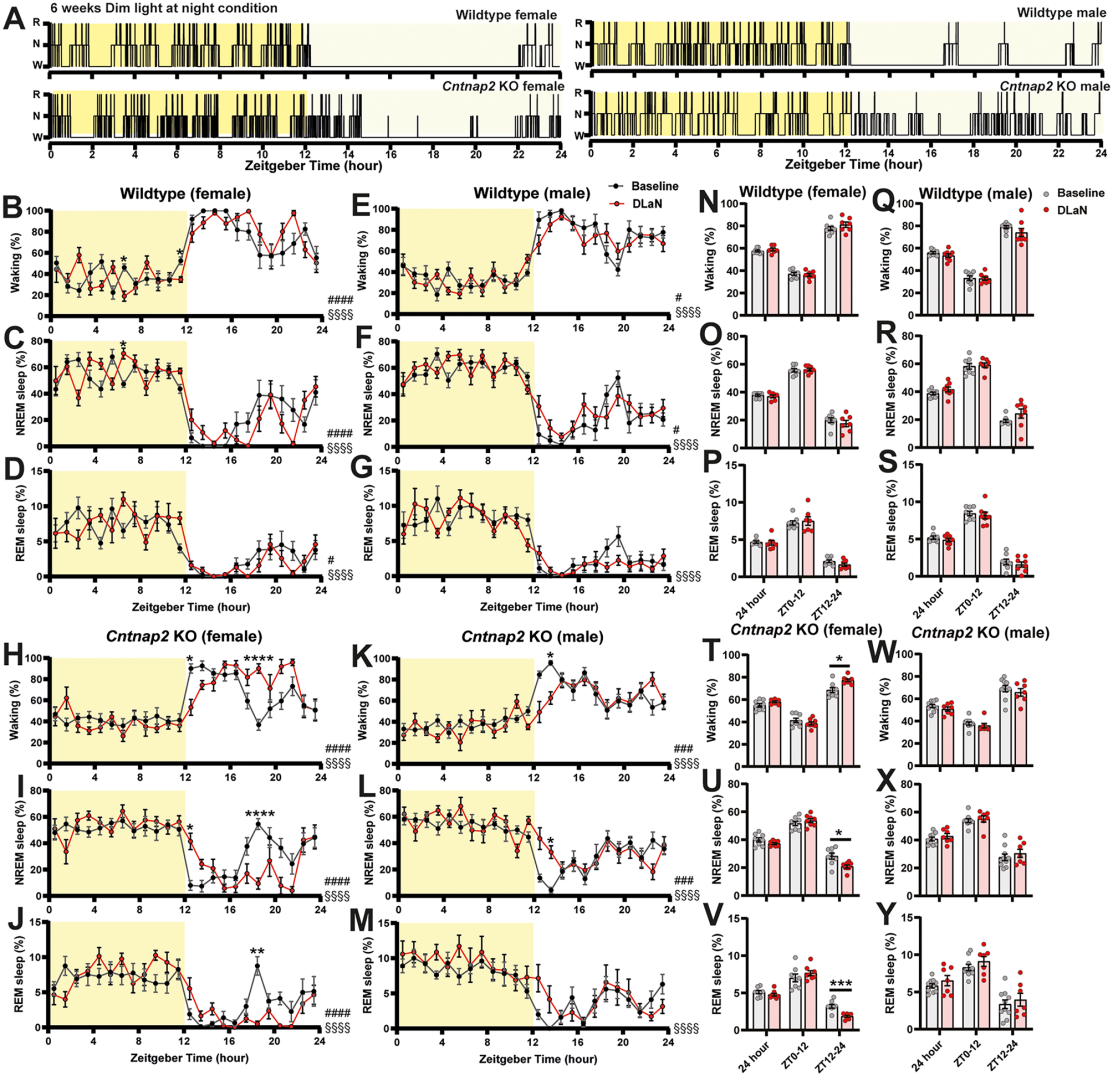
Sleep architecture of WT and *Cntnap2* KO mice during baseline and 6 weeks of DLaN. (**A**) Representative 24-hrs hypnograms. Bright yellow indicates the light phase, and light yellow indicates the dim light at night. (**B-M**) Twenty-four-hour distributions of time spent in wakefulness, NREM sleep, and REM sleep for WT females (**B-D**), WT males (**E-G**), KO females (**H-J**) mice and KO males (**K-M**) mice during baseline (black circle) and after 6 weeks of DLaN (red circle). Pound sign (#) indicates a significant interaction between the factors “zeitgeber time” and “DLaN” (two-way repeated-measures ANOVA with Geisser-Greenhouse’s correction; #*p* < 0.05, ###*p* < 0.001, ####*p* < 0.0001). Main effect of “zeitgeber time” and “DLaN” were showed as “§” and “∆” (§§§§*p* < 0.0001). Asterisks in panel B-M indicate significant post-hoc differences between baseline and 6 weeks of DLaN (Bonferroni multiple comparisons test, *p < 0.05, **p < 0.01, ****p < 0.0001). (**N-Y**) Time spent in wakefulness, NREM sleep and REM sleep over 24 h, in the light and in the dark periods for WT females (**N-P**), WT males (**Q-S**), KO females (**T-V**) mice and KO males (**W-Y**) mice during baseline (grey circle) and after 6 weeks of DLaN (red circle). Asterisks in panels T-V indicate significant differences between baseline and DLaN (paired t-test or Wilcoxon matched-pairs signed rank test, *p < 0.05, ***p < 0.001). Sample size: WT female, baseline = 8 and DLaN = 7; WT male, baseline = 8 and DLaN = 8; KO female, baseline = 8 and DLaN = 8; KO male, baseline = 10 and DLaN = 7. Data are shown as mean ± SEM

Most previous studies on the effect of DLaN were performed in male rodents. Here, we observed distinct sex differences in sleep patterns under DLaN exposure in both WT and KO mice (Fig. 2; Tables 1 and 2). Analysis of total 24-hr sleep revealed a significant sex difference in wake and REM sleep, with KO females showing 7.2 ± 2.0% more wake compared to KO males. REM sleep differed significantly across genotypes and sexes, with KO males exposed to 6 weeks of DLaN displaying a higher total amount of REM sleep than both KO females and WT males (Table 2). Light-dark phase analyses further highlighted sex-dependent effects. During the light phase, genotype significantly influenced NREM sleep, whereas sex significantly affected REM sleep. During the dim light phase, both genotype and sex significantly influenced sleep patterns: KO females exhibited increased wake compared to KO males, while KO males had significantly more REM sleep than KO females and WT males (Table 2). Interestingly, DLaN exposure did not affect episode durations or frequency in female mice. In contrast, male mice exposed to 6 weeks of DLaN, episode duration of waking was extended relative to baseline conditions, as were NREM and REM sleep episode durations (Supplemental Fig. 2). These results suggest that DLaN exerts a stronger impact on sleep regulation in a sex-dependent manner, particularly in KO mice.

**Table 2:** Time spent in three vigilance States in WT and *Cntnap2* KO mice under LD and DLaN. Data shown as means ± SEM. A genotype difference compared in the same sex; b sex difference compared in the same genotype

Baseline	WT female	WT male	KO female	KO male	Genotype	Sex
24 h Waking (%)	57.82 ± 0.29	55.99 ± 0.34	54.92 ± 0.55	53.45 ± 0.46	*F*_(1, 30)_ = 4.458; ***p*** **= 0.0432**	*F*_(1, 30)_ = 1.645
24 h NREM sleep (%)	37.44 ± 0.27	38.83 ± 0.34	39.97 ± 0.54	40.72 ± 0.42	*F*_(1, 30)_ = 3.304	*F*_(1, 30)_ = 0.783
24 h REM sleep (%)	4.74 ± 0.05	5.18 ± 0.08	5.11 ± 0.08	5.82 ± 0.09	*F*_(1, 30)_ = 4.823; ***p*** **= 0.0359**	*F*_(1, 30)_ = 6.146; ***p*** **= 0.0190**
Light phase Waking (%)	37.72 ± 0.55	33.27 ± 0.75	41.35 ± 0.68	37.86 ± 0.45	*F*_(1, 30)_ = 5.446; ***p*** **= 0.0265**	*F*_(1, 30)_ = 5.089; ***p*** **= 0.0315**
Light phase NREM sleep (%)	54.89 ± 0.54	58.30 ± 0.71	51.65 ± 0.57	53.85 ± 0.41	*F*_(1, 30)_ = 5.708; ***p*** **= 0.0234**	*F*_(1, 30)_ = 3.049
Light phase REM sleep (%)	7.39 ± 0.09	8.43 ± 0.11	7.01 ± 0.18	8.29 ± 0.13	*F*_(1, 30)_ = 0.449	*F*_(1, 30)_ = 8.875; ***p*** **= 0.0057**
Dark phase Waking (%)	77.91 ± 0.65	78.70 ± 0.49	68.50 ± 0.80 ^a^	69.05 ± 0.90 ^a^	*F*_(1, 30)_ = 17.44; ***p*** **= 0.0002**	*F*_(1, 30)_ = 0.087
Dark phase NREM sleep (%)	20.00 ± 0.60	19.36 ± 0.39	28.28 ± 0.76 ^a^	27.59 ± 0.74 ^a^	*F*_(1, 30)_ = 17.45; ***p*** **= 0.0002**	*F*_(1, 30)_ = 0.113
Dark phase REM sleep (%)	2.09 ± 0.06	1.93 ± 0.13	3.22 ± 0.09	3.36 ± 0.18	*F*_(1, 30)_ = 9.502; ***p*** **= 0.0044**	*F*_(1, 30)_ = 0.001
**6 weeks of DLaN**						
24 h Waking (%)	58.57 ± 0.46	53.41 ± 0.59	57.95 ± 0.26 ^b^	50.74 ± 0.69	*F*_(1, 26)_ = 1.340	*F*_(1, 26)_ = 19.00; ***p*** **= 0.0002**
24 h NREM sleep (%)	36.87 ± 0.39	41.71 ± 0.60	37.28 ± 0.24	42.73 ± 0.70	*F*_(1, 26)_ = 0.2690	*F*_(1, 26)_ = 13.76; ***p*** **= 0.0010**
24 h REM sleep (%)	4.56 ± 0.12	4.86 ± 0.08	4.77 ± 0.06	6.53 ± 0.26 ^a b^	*F*_(1, 26)_ = 6.789; ***p*** **= 0.0235**	*F*_(1, 26)_ = 7.050; ***p*** **= 0.0134**
Light phase Waking (%)	36.22 ± 0.52	32.71 ± 0.53	38.63 ± 0.50	35.83 ± 0.76	*F*_(1, 26)_ = 3.055;	*F*_(1, 26)_ = 3.978
Light phase NREM sleep (%)	56.28 ± 7.50	59.14 ± 8.15	53.71 ± 0.50	55.06 ± 0.85	*F*_(1, 26)_ = 4.414; ***p*** **= 0.0455**	*F*_(1, 26)_ = 1.770
Light phase REM sleep (%)	7.50 ± 0.22	8.15 ± 0.16	7.66 ± 0.12	9.11 ± 0.25	*F*_(1, 26)_ = 1.244	*F*_(1, 26)_ = 4.361; ***p*** **= 0.0467**
Dim light phase Waking (%)	80.93 ± 0.90	74.11 ± 1.24	77.28 ± 0.42 ^b^	65.64 ± 1.27	*F*_(1, 26)_ = 4.804; ***p*** **= 0.0375**	*F*_(1, 26)_ = 11.15; ***p*** **= 0.0025**
Dim light phase NREM sleep (%)	17.45 ± 0.82	24.28 ± 1.13	20.86 ± 0.40	30.40 ± 1.11	*F*_(1, 26)_ = 3.661	*F*_(1, 26)_ = 10.80; ***p*** **= 0.0029**
Dim light phase REM sleep (%)	1.62 ± 0.08	1.57 ± 0.12	1.87 ± 0.05	3.95 ± 0.33 ^a b^	*F*_(1, 26)_ = 8.160; ***p*** **= 0.0083**	*F*_(1, 26)_ = 4.901; ***p*** **= 0.0358**

While the above analyses provide an integrated view of daily (24-h) and phase-specific (12-h) changes, examining sleep at finer temporal resolution revealed how these effects evolved over time within the day. After just 2 weeks of DLaN, only KO females exhibited reduced NREM and REM sleep during the dark phase, whereas KO males and WT mice did not show significant differences (Supplemental Fig. 1). After 6 weeks of exposure, the DLaN effects became more pronounced and extended to other groups. Representative hypnograms revealed that after 6 weeks DLaN, both male and female KO mice exhibited dramatic alterations in sleep architecture (Fig. 2A). In KO females, DLaN delayed waking time around the light-dark transition and increased dim light phase wakefulness (Fig. 2H, Zeitgeber time x DLaN: *F*_23, 322_ = 3.781, *p* < 0.0001; **2T**, t_7_ = 3.388, *p* = 0.0116). Correspondingly, nighttime NREM sleep (Fig. 2I, Zeitgeber time x DLaN: *F*_23, 322_ **=** 3.754; **2U**, t_7_ = 5.950, *p* = 0.0006) and REM sleep (Fig. 2J, Zeitgeber time x DLaN: *F*_23, 322_ **=** 2.878; **2 V**, t_7_ = 2.959, *p* = 0.0211) were significantly reduced. In KO males, DLaN decreased wake and increased in NREM sleep around the light-dark transition (Fig. 2K, L). WT mice exhibited less pronounced changes than KO mice (Fig. 2), but two-way repeated measures ANOVA revealed significant interactions between DLaN and Zeitgeber time in all states in WT females (wakefulness: *F*_23, 299_ = 2.840, *p* < 0.0001; NREM sleep: *F*_23, 299_ = 2.899, *p* < 0.0001; REM sleep: *F*_23, 299_ = 1.768, *p* = 0.018), and in wakefulness in WT males (*F*_23, 322_ = 1.632, *p* = 0.035), indicating that DLaN altered the temporal distribution of sleep–wake rhythm even in WT mice. These findings suggest that KO males and females may exhibit distinct phenotypes under chronic DLaN exposure, reflecting differential susceptibility to DLaN induced sleep disruption.

### Power spectrum of NREM sleep and the slow wave activity under DLaN

**Fig. 3:**
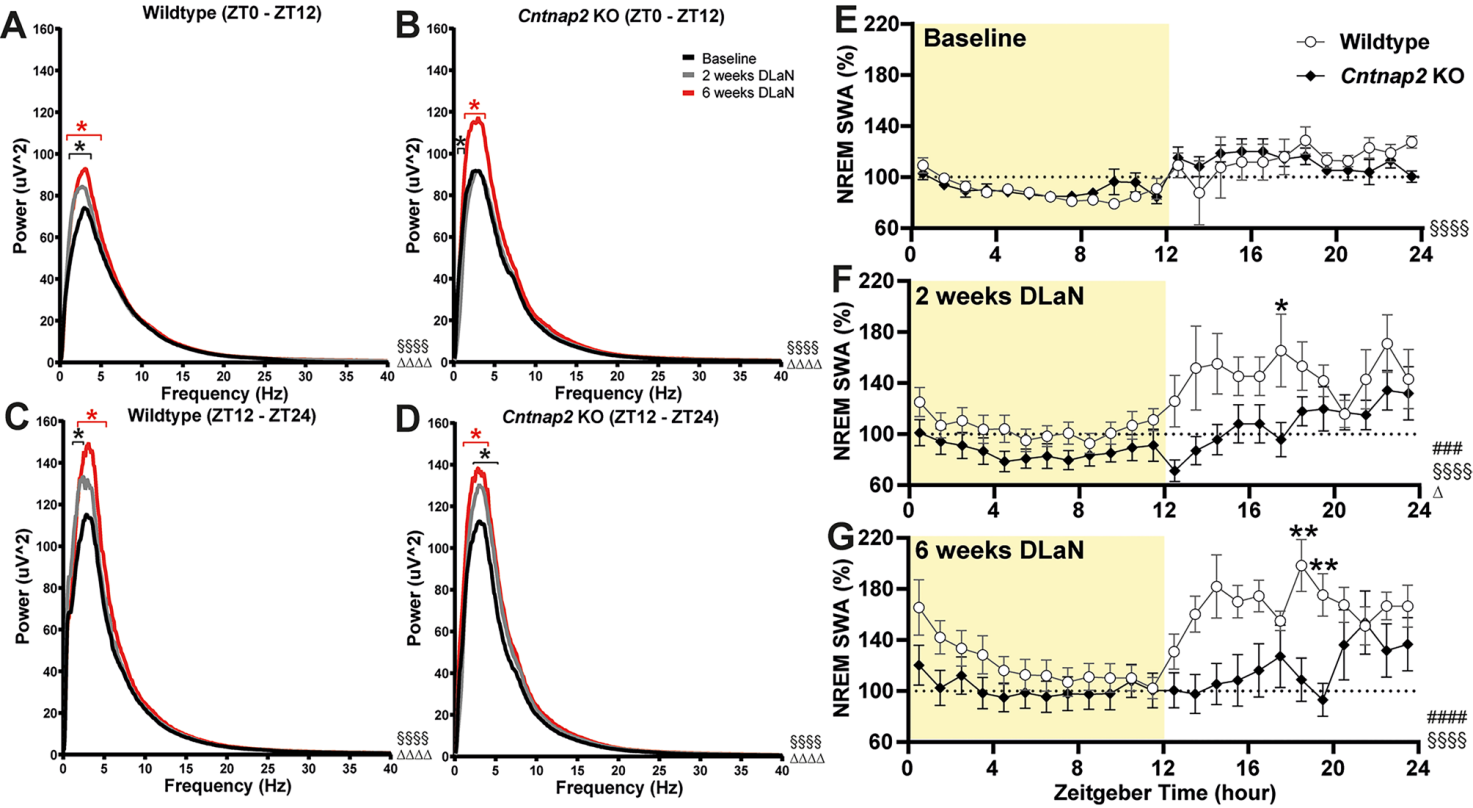
EEG spectrum and slow wave activity in NREM sleep of WT and KO mice at baseline and after DLaN. (**A-D**) Absolute EEG power spectra during NREM sleep in the light phase (**A**,** B**) and dark phase (**C**,** D**) for baseline (black), 2 weeks of DLaN (gray), and 6 weeks of DLaN (red) in WT and KO mice. Main effect from two way ANOVA of “Frequency” and “DLaN” were showed as “§” and “∆” (§§§§*p* < 0.0001, ∆∆∆∆*p* < 0.0001). Black and red asterisks in panel A-D indicate significant post-hoc differences between baseline and 2 weeks or 6 weeks of DLaN (Bonferroni multiple comparisons test, * *p* = 0.05–0.0001). The frequency bin size is 0.1 Hz. Data are shown as the mean values. (**E-G**) Time course of slow-wave activity in NREM sleep for WT (white) and KO mice (black) under baseline LD conditions (**E**), after 2 weeks (**F**), and after 6 weeks (**G**) of DLaN. Pound sign (#) indicates a significant interaction between the factors “zeitgeber time” and “DLaN” (mixed-effects model (REML) with Geisser-Greenhouse’s correction; ###*p* < 0.001, ####*p* < 0.0001). Main effect of “zeitgeber time” and “DLaN” were showed as “§” and “∆” (§§§§*p* < 0.0001, ∆*p* < 0.05). Asterisks in panel E-G indicate significant post-hoc differences between WT and KO mice (Bonferroni multiple comparisons test, *p < 0.05, **p < 0.01). *n* = 11–16 mice per genotype and condition

The NREM sleep power spectrum revealed a significant increase in the delta range following DLaN exposure (Fig. 3). In WT mice, the absolute power in the delta range of NREM sleep increased over time, with a significant elevation observed after both 2 and 6 weeks of DLaN exposure compared to baseline LD conditions in the light phase (DLaN effect: *F*_2, 14800_ = 80.43, *p* < 0.0001, 2 weeks DLaN: 1.0–3.8 Hz, *p* < 0.05, 6 weeks DLaN: 0.8–5.1 Hz, *p* < 0.05; Fig. 3A, DLaN effect: *F*_2, 14400_ = 51.72, *p* < 0.0001, 2 weeks DLaN: 1.0–2.5 Hz, *p* < 0.05, 6 weeks DLaN: 1.6–5.3 Hz, *p* < 0.05, Fig. 3C). Similarly, KO mice exhibited a relatively smaller but significant increase in absolute power in the delta range (DLaN effect: *F*_2, 16800_ = 46.89, *p* < 0.0001, 2 weeks DLaN: 0.4–1.1 Hz, *p* < 0.05, 6 weeks DLaN: 1.2–4.1 Hz, *p* < 0.05; Fig. 3B, DLaN effect: *F*_2, 16800_ = 47.45, *p* < 0.0001, 2 weeks DLaN: 2.5–5.2 Hz, *p* < 0.05, 6 weeks DLaN: 1.5–4.1 Hz, *p* < 0.05, Fig. 3D). To further examine the spectral changes induced by DLaN, we analyzed the EEG power spectrum during wake and REM sleep, but these changes were relatively minor. In KO mice, there was a slight increase in the theta range during REM sleep during the light phase and in WT mice, a small increase was observed in the theta range during REM sleep in the dark phase (Supplemental Fig. 3).

Given these observed changes in the NREM sleep power spectrum within the delta range, we further analyzed the relative slow wave activity (SWA) during NREM sleep over 24 h, as SWA serves as a well-established marker of sleep homeostasis and sleep pressure. Under baseline LD conditions, there were no significant interaction between Genotype and Zeitgeber time or main Genotype effect in SWA (Genotype x Zeitgeber time: *F*_23, 546_ = 1.313, *p* = 0.151, Genotype effect: *F*_1, 546_ = 0.018, *p* = 0.894, Fig. 3E). However, after 2 weeks of DLaN exposure, SWA increased significantly more in WT mice than in KO mice, particularly during the dark phase (Genotype x Zeitgeber time: *F*_23, 464_ = 2.152, *p* = 0.0016, Genotype effect: F _1, 24_ = 4.895, *p* = 0.037, Fig. 3F). This pattern persisted with extended exposure, with significant differences observed in WT mice at 6 weeks (Genotype x Zeitgeber time: *F*_23, 448_ = 4.151, *p* < 0.0001, Genotype effect: *F*_1, 23_ = 2.543, *p* = 0.124, Fig. 3G). These findings demonstrate that chronic DLaN selectively enhances NREM delta power, with a more pronounced and sustained impact in WT mice, underscoring genotype-dependent differences in the homeostatic response to nocturnal light exposure.

### Abnormal EEG events increased after DLaN exposure in *Cntnap2* KO mice

**Fig. 4:**
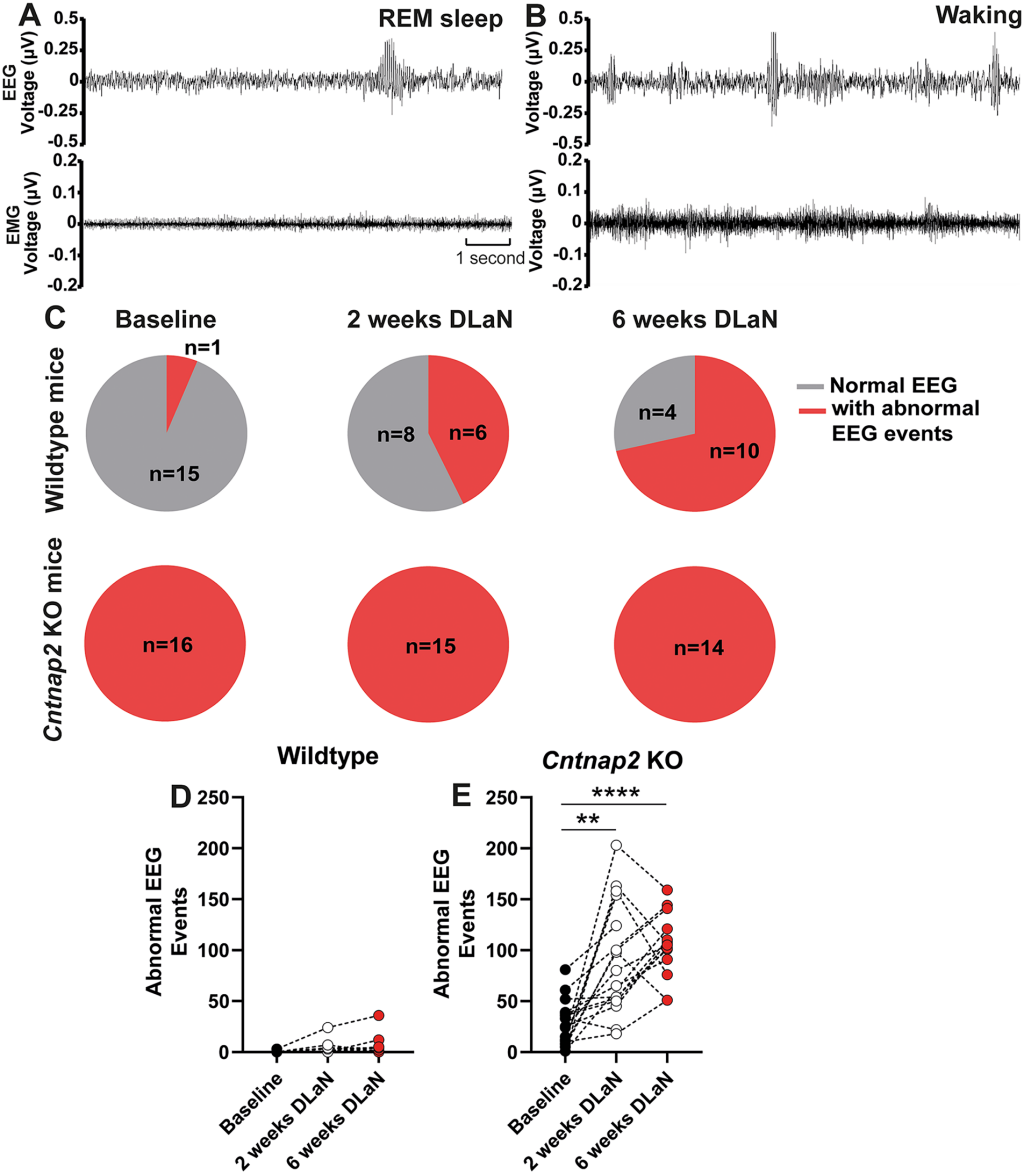
*Cntnap2* KO mice exhibit abnormal EEG events. (**A-B**) Representative spike-like activity during REM sleep (**A**) and wakefulness (**B**) shown with electroencephalogram and electromyogram recordings. (**C**) Number of mice exhibiting abnormal EEG events during baseline, after 2 weeks and 6 weeks of DLaN in WT and *Cntnap2* KO mice. In each pie chart, the grey portion (with numbers) indicates the number of mice showing normal EEG, and the red portion (with numbers) indicates the number of mice showing abnormal EEG events. (**D-E**) Total number of abnormal EEG events during baseline, 2 weeks and 6 weeks of DLaN in WT mice and KO mice. Asterisks indicate significant differences between baseline and DLaN (**p = 0.0048, ****p < 0.0001, Bonferroni multiple comparisons test following a mixed-effects model (REML) with Geisser-Greenhouse’s correction). *n* = 14–16 mice per genotype and condition. Data are shown as individual values

During EEG scoring, abnormal bursts of high-amplitude, ‘hypersynchronized’ EEG activity were observed in *Cntnap2* KO mice during both REM sleep (Fig. 4A) and wake (Fig. 4B). This abnormal EEG activity, was previously reported in *Cntnap2* KO mice [34] and was referred to as seizure-like activity. However, since this event is not associated with behavioral seizures as measured by the EMG, we will refer this as “abnormal EEG event” to more accurately reflect its nature [34]. We quantified the occurrence of these abnormal EEG events under three conditions in both WT and KO mice. At baseline, all 16 *Cntnap2* KO mice exhibited abnormal EEG events (Figs. 4C and 100%), and these persisted under DLaN exposure. *Cntnap2* KO mice showed a significant increase in abnormal EEG events following DLaN exposure compared to baseline (*F*_1.4, 18.91_= 20.40, *p* < 0.0001, mean ± SEM: baseline: 26.59 ± 5.49 events; 2 weeks DLaN: 92.47 ± 14.62 events; 6 weeks DLaN: 104.8 ± 8.43 events, Fig. 4E). In contrast, in WT mice, only a single abnormal EEG event was observed at baseline. Following 2 weeks of DLaN exposure, 6 out of 14 WT mice (42.9%) exhibited abnormal EEG events, increasing to 10 out of 14 WT mice (71.4%) after 6 weeks. Despite this increase in incidence, the frequency of abnormal EEG events in WT mice did not significantly differ between LD and DLaN conditions (*F*_1.279, 16.63_ = 2.282, *p* = 0.1459, Fig. 4D).

### The daily modulation and EEG power spectrum of the abnormal EEG events in *Cntnap2* KO mice

**Fig. 5:**
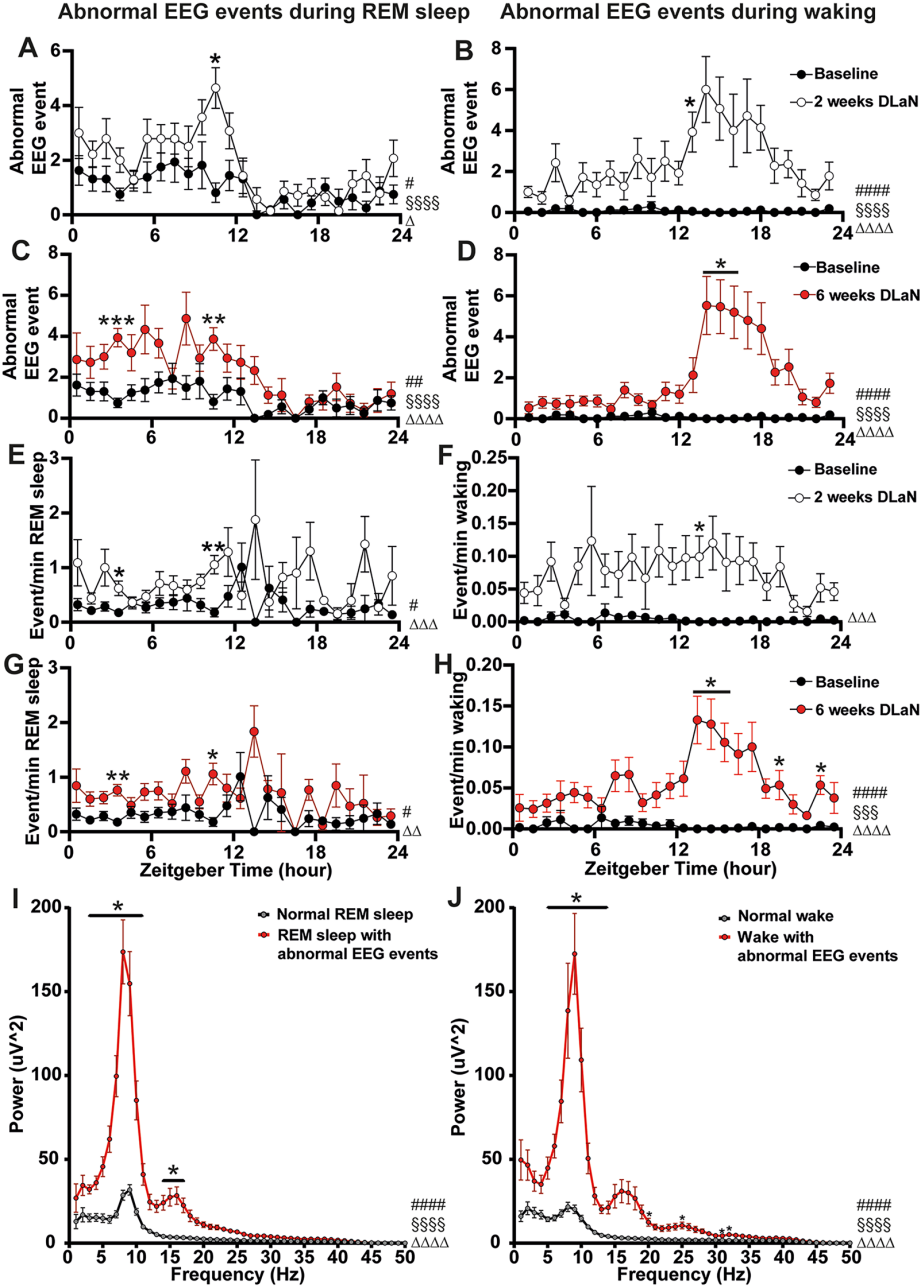
Daily distribution and power spectrum analysis of abnormal EEG events in *Cntnap2* KO mice. (**A-H)** Time course of occurrence and quantified occurrence during REM sleep (**A**, **C**, **E**, **G**) and wakefulness (**B**,** D**,** F**,** H**) in KO mice under baseline (grey), after 2 weeks (**A**, **B**,** E**,** F**, white), and after 6 weeks (**C**, **D**, **G**, **H**, red) of DLaN. Pound sign (#) indicates a significant interaction between the factors “zeitgeber time” and “DLaN” (Two-way repeated measures ANOVA or mixed-effects model [REML] with Geisser-Greenhouse’s correction; #*p* < 0.05, ##*p* < 0.01, ####*p* < 0.0001). Main effect of “Zeitgeber time” and “DLaN” were showed as “§” and “∆” (§§§*p* < 0.001, §§§§*p* < 0.001, ∆*p* < 0.05, ∆∆*p* < 0.01, ∆∆∆*p* < 0.001, ∆∆∆∆*p* < 0.0001). Asterisks indicate significant post-hoc differences between baseline and DLaN (Bonferroni multiple comparisons test, * *p* < 0.05). *n* = 14–16 mice per condition. (**E-F**) Absolute EEG power spectrum during REM sleep (**I**) and wakefulness (**J**) for abnormal EEG event (red) and normal EEG (black) in KO mice after 6 weeks of DLaN. Pound sign (#) indicates a significant interaction between the “abnormal EEG events” and “frequency” (Two-way repeated measures ANOVA with Geisser-Greenhouse’s correction; ####*p* < 0.0001). Main effect of “frequency” and “abnormal EEG events” were showed as “§” and “∆” (§§§§*p* < 0.001, ∆∆∆∆*p* < 0.0001). Asterisks indicate significant differences between abnormal EEG and normal EEG (Bonferroni multiple comparisons test, * *p* < 0.05). The frequency bin size is 1 Hz. Data are shown as mean ± SEM

Since the abnormal EEG events occurred during both REM sleep and waking, we quantified their distribution over 24 h under baseline and post-DLaN conditions in KO mice. At baseline, these events were infrequent during wakefulness and occurred predominantly during REM sleep. After 2 weeks of DLaN exposure, there was a significant increase in abnormal EEG events during REM sleep at ZT11 (Zeitgeber time x DLaN: *F*_23, 666_ = 1.617, *p* = 0.035, DLaN: *F*_1, 29_ = 6.620, *p* = 0.016, Fig. 5A). Following 6 weeks of DLaN exposure, abnormal EEG events during REM sleep became more pronounced during the light phase (Zeitgeber time x DLaN: *F*_23, 644_ = 2.091, *p* = 0.0022, DLaN: *F*_1, 28_ = 19.58, *p* = 0.0001, Fig. 5C). Similarly, abnormal EEG events during wakefulness increased significantly after 2 weeks of DLaN exposure, particularly in the active phase (Zeitgeber time x DLaN: *F*_23, 667_ = 3.532, *p* < 0.0001, DLaN: *F*_1, 29_ = 19.51, *p* = 0.0001, Fig. 5B) and remained elevated after 6 weeks of DLaN (Zeitgeber time x DLaN: *F*_23, 644_ = 7.988, *p* < 0.0001, DLaN: *F*_1, 28_ = 48.63, *p* < 0.0001, Fig. 5D).

The observed daily rhythm in abnormal EEG events may be influenced by daily modulation in wakefulness and REM sleep. To account for this, we normalized the frequency of abnormal EEG events by calculating their occurrence per minute of wakefulness or REM sleep. After 2 weeks of DLaN exposure, there was a significant effect of DLaN in abnormal EEG events during both REM sleep (*F*_1, 29_ = 15.07, *p* = 0.0006, Fig. 5E) and wake (*F*_1, 29_ = 18.78, *p* = 0.0002, Fig. 5F), with neither a strong significant interaction between time and DLaN (REM sleep: *F*_23, 488_ = 1.570, *p* = 0.046, Wakefulness: *F*_23, 667_ = 1.255, *p* = 0.190) nor a main effect of time (REM sleep: *F*_23, 488_ = 1.462, *p* = 0.0774, Wake: *F*_4.226, 122.6_ = 1.171, *p* = 0.327). This suggests that DLaN increased abnormal EEG activity independently of time-of-day without altering its temporal distribution. After 6 weeks of DLaN exposure, both DLaN and interaction of DLaN and time had a significant effect on abnormal EEG events during REM sleep, though no effect of time was observed (Zeitgeber time x DLaN: *F*_23, 472_ = 1.629, *p* = 0.034, Zeitgeber time: *F*_4.438, 91.08_ = 1.969, *p* = 0.099, DLaN: *F*_1, 28_ = 12.44, *p* = 0.0015, Fig. 5G). Conversely, abnormal EEG events during waking showed a significant interaction, and main effect of time and DLaN (Zeitgeber time x DLaN: *F*_23, 644_ = 4.186, *p* < 0.0001, Zeitgeber Time: *F*_7.350, 205.8_ = 3.648, *p* = 0.0008, DLaN: *F*_1, 28_ = 47.05, *p* < 0.0001, Fig. 5H), indicating the emergence of a daily rhythmic pattern after prolonged exposure.

Spectral analysis of abnormal EEG events after 6 weeks of DLaN exposure revealed a substantial increase in power density in the theta range, with an additional increase in the 14–17 Hz range during REM sleep (Abnormal EEG x Frequency: *F*_49, 1176_ = 33.86, *p* < 0.0001, Frequency: *F*_49, 1176_ = 73.11, *p* < 0.0001, Abnormal EEG: *F*_1, 24_ = 49.38, *p* < 0.0001, 4–11 Hz, 14–17 Hz, *p* < 0.05, Fig. 5I; Abnormal EEG x Frequency: *F*_49, 1176_ = 19.16, *p* < 0.0001, Frequency: *F*_3.01, 72.24_ = 35.53, *p* < 0.0001, Abnormal EEG: *F*_1, 24_ = 43.61, *p* < 0.0001, 5–13 Hz, 20, 25, 32, 33 Hz, *p* < 0.05, Fig. 5J). This increased theta power was a consistent feature across the abnormal EEG events under both LD and DLaN conditions (Supplemental Fig. 4).

## Discussion

There is growing evidence that nightly exposure to light extracts a toll on our health and wellness [38, 39]. Concerns about exposure to DLaN may be particularly relevant to individuals with neurodevelopmental disorders who are vulnerable to environmental perturbations. To explore this issue and its underlying mechanisms, we examined the impact of DLaN on the *Cntnap2* KO model of neurodevelopmental disorders. In prior work, we showed that DLaN disrupts rhythms in activity and sleep behavior in the *Cntnap2* KO mice [36, 37]. These disruptions correlated with DLaN-driven reductions in social behavior and increases in repetitive behaviors. While video analysis of sleep is useful for long-term, non-invasive tracking, it lacks the physiological precision of EEG, which is essential for defining sleep stages and quality. In the present study, we used EEG recordings to assess vigilance states (wake, NREM, and REM sleep) and examine the frequency of abnormal EEG events in the ASD model. Additionally, we explored potential sex differences, as prior work has largely focused on males.

### Sleep disruptions in *Cntnap2* KO mice under baseline conditions

*Cntnap2* is a gene that is associated with both ASD and CDFE syndrome, and sleep problems are widely recognized in both patient populations. Sleep disruptions have been reported in other ASD mouse models, including Neuroligin-2 knockout (*Nlgn2* KO) and Neuroligin-3 knockout (*Nlgn3* KO) mice [40, 41]. The BTBR line as well as the fragile X mental retardation syndrome 1 (*Fmr1*) KO and BTBR mice show genotype-time interactions [42] and evidence for sleep fragmentation in the *Fmr1* KO [43]. These variations in sleep phenotypes likely stem from differences in genetic mutations leading to distinct synaptic and cellular dysfunctions [44]. Under baseline conditions, all mice exhibited robust daily rhythms in wake, NREM sleep, and REM sleep, and the vigilance states were affected by the interaction between time and genotype, although only REM sleep was significantly influenced by genotype. Prior work also found that immobility defined sleep behavior was relatively unaffected in the *Cntnap2* KO [36], but EEG-based sleep analysis revealed fragmented wakefulness and blunted state rhythms in the *Cntnap2* KO mice [34]. The EEG based fragmentation is consistent with our findings, demonstrating that sleep fragmentation is a robust feature of the *Cntnap2* KO model. Both male and female KO mice exhibited increased fragmentation of wake, NREM, and REM sleep. These findings contrast with the *Nlgn2* KO mice, which exhibit more consolidated sleep compared to WT, highlighting model-specific sleep phenotypes [41]. In the present study, both sexes of KO mice exhibited a striking increase in vigilance state fragmentation, decreased waking and increased sleep during the night, a pattern that may reflect underlying deficits in circadian regulation.

### *Cntnap2* KO mice showed dramatic changes in sleep architecture after exposure to DLaN

Under DLaN, WT mice did not show significant alterations in vigilance states even after six weeks of exposure. These findings are consistent with a previous study demonstrating that chronic exposure to 5 lx nighttime light did not significantly alter the sleep structure of male WT mice [45]. Another study reported altered daily rhythm of vigilance state under DLaN, particularly in REM sleep [46]. In contrast, *Cntnap2* KO mice showed disrupted sleep-wake rhythms after six weeks of DLaN exposure. Both male and female KO mice exhibited delayed wake onset during the active phase, suggesting a similar delay effect across sexes. This delayed sleep/wake onset mirrors findings from prior human and animal studies examining the impact of nighttime light exposure [47–49].

Under DLaN, sex-specific differences emerged in *Cntnap2* KO mice. Female KO mice showed more pronounced alterations in sleep architecture, marked by increased wake, suggesting that females may be more affected to the disruptive effects of DLaN. In contrast, male KO mice displayed more consolidated bouts of wake, NREM, and REM sleep under DLaN, whereas female KO and WT mice showed no significant changes in sleep architecture. These divergent responses may be shaped by hormonal influences. Importantly, given the male bias in ASD research [50], the inclusion of both sexes in this study allowed us to detect subtle but significant sex-dependent effects of DLaN. Similar sex-specific responses to nighttime light have been reported in other species, such as birds, where females exhibit greater wakefulness during nocturnal light exposure [51]. Together, these findings underscore the role of both environmental and hormonal factors in shaping sex-dependent sleep phenotypes under DLaN conditions.

Spectral analysis of the EEG during NREM sleep revealed a significant increase in delta power in both WT and KO mice under DLaN compared to baseline LD conditions, indicating elevated sleep pressure. This finding aligns with prior studies reporting similar effects after chronic DLaN exposure for durations ranging from one week to one month [47]. A 24-hr profile of slow-wave activity (SWA) data demonstrated more pronounced changes during the dark phase in WT mice. Although KO mice exhibited increased delta power in their NREM sleep power spectrum, these mice also had increased sleep during the dim light phase which may have resulted in decreased relative SWA. In contrast, the extended waking periods in WT mice likely contributed to their elevated SWA. Both spectral data and relative SWA data suggest that KO and WT mice experienced greater sleepiness under DLaN conditions compared to baseline LD conditions.

### Abnormal EEG activity occurrence increased under DLaN conditions

Epileptic seizures and abnormal EEG patterns have been previously observed in *Cntnap2* KO mice [30, 34]. Consistent with previous findings, we observed abnormal electrical discharges in both male and female KO mice, indicating that this EEG feature is present across sexes. Under baseline conditions these abnormal EEG events occurred primarily during REM sleep and were rarely observed during waking and never during NREM sleep. Although approximately half of all seizures occur during sleep, most sleep-related seizures are associated with NREM sleep, with REM-related seizures being relatively rare [52]. Similarly, *Ank3-exon1b* knockout (Ank3-1b KO) mice, a model relevant to epilepsy, also exhibited a higher prevalence of seizures during REM sleep [53], and *Nlgn2* KO mice also showed abnormal EEG events that predominated during wakefulness and REM sleep [54]. These findings suggest that abnormal EEG activity in epilepsy and autism models is strongly associated with REM sleep.

As observed in patients, seizures often occur at specific times of day, and the circadian rhythm of seizure occurrence varies across epilepsy subtypes and seizure foci [18, 55, 56]. In *Nlgn2* KO mice, hypersynchronized ECoG events peak around ZT 9–10, whereas in *Cntnap2* KO mice, abnormal EEG event density is highest at the onset of the active phase under LD conditions. In the present study, chronic DLaN exposure significantly increased the frequency of abnormal EEG events in KO mice during both REM sleep and wake. After six weeks of DLaN, abnormal EEG event density displayed a distinct rhythmic pattern in wake, suggesting that prolonged nighttime light exposure may trigger adaptive network mechanisms. Importantly, WT mice also exhibited abnormal EEG events under DLaN, albeit at lower frequency, indicating that DLaN itself may act as an environmental risk factor capable of promoting hyperexcitability even in the absence of genetic vulnerability.

### Excitatory/inhibitory imbalance in *Cntnap2* KO mice

Prior work suggests that network-wide differences from WT might underlie the increased occurrence of abnormal EEG events in *Cntnap2* KO mice. *Cntnap2* expression is highest in cortical and striatal regions [57], and its loss leads to dysregulated inhibitory synaptic transmission, and impaired neuronal migration. Power spectral analysis revealed that these abnormal EEG events were characterized by elevated theta power, which is often generated by the hippocampus suggesting that the hippocampus may play a critical role in the generation of these abnormal EEG events. A reduction in parvalbumin-positive interneurons in the hippocampus has been described in *Cntnap2* KO mice [30]. Dysfunction of parvalbumin-positive (PV+) interneurons has been implicated in several neurological disorders, including epilepsy [58, 59]. PV interneuron failure, potentially via depolarization block and impaired inhibitory synaptic function, results in a reduction in overall inhibition and ultimately in seizures or abnormal EEG event in the *Cntnap2* KO mice.

In addition to PV neuron dysfunction, *Cntnap2* KO animals have been reported to exhibit reduced functional synaptic connectivity and decreased synapse density in the medial prefrontal cortex [60, 61]. Notably, modulation of the excitatory/inhibitory (E/I) balance in the prefrontal cortex has been shown to rescue social behavior deficits in *Cntnap2* KO mice [62]. A recent study demonstrated that the daily E/I balance was disrupted in *Fmr1* KO and BTBR mice, two other autism mouse models [42], suggesting that disrupted daily E/I balance may be a common feature of autism-related models. Environmental factors, such as DLaN, may further exacerbate these network imbalances. Chronic exposure to 5 lx DLaN has been shown to alter thalamo-cortical neuronal networks in animal studies [47]. In general these findings suggest that even low-level nighttime light can impair neural homeostasis and synaptic regulation [63]. Given that DLaN can disrupt brain networks, and *Cntnap2* KO mice already exhibit disrupted E/I balance, exposure to DLaN likely intensifies these pre-existing neural deficits. This compounded disruption may explain the increased occurrence of abnormal EEG event observed in KO mice under DLaN conditions, as compared to the baseline LD condition.

### Disrupted circadian rhythm is a risk factor for abnormal EEG event occurrence in *Cntnap2* KO mouse model

The circadian clock drives daily rhythms in physiology through a variety of mechanisms. In the mouse cortex, the majority of synaptic transcripts exhibit daily rhythms in abundance that peak before dawn and dusk [64] and robust rhythms in phosphoproteins have been described [65]. In prior work, we have shown that DLaN altered the phase and amplitude of the molecular clock expressed in the *Cntnap2* KO in a tissue-specific manner [36] suggesting likely downstream effects on cortical physiology. Interactions between disruption of the circadian clock and epilepsy has been suggested by earlier work. For example, mutations in the RORα gene (RORA) link to intellectual disability including autistic symptoms [66]. In another example, mutations in SCN1A which encodes a voltage-gated sodium channel (Nav1.1), which is necessary for normal circadian rhythms, and is a risk factor for epilepsy [67, 68]. Similarly, a Nav1.1 mutation causing reduced interneuron excitability and seizures also causes sleep impairment in a mouse model of Dravet Syndrome [69].

The evidence for bi-directional associations between circadian disruption and seizure susceptibility is compelling. DLaN is a mild environmental disruptor of circadian rhythms and one that may be commonly experienced by patients with ASD and other neurodevelopmental conditions. Our work raises the possibility that environmental light exposure may exacerbate core features of developmental disorders, including disrupted sleep and heightened seizure susceptibility. Moreover, our results raise the possibility of using circadian medicine to develop new therapeutic approaches. Many have argued the possible benefits of interventions at specific times of the day to strengthen the patients’ circadian rhythms. In this mouse model, we have already shown that nighttime treatment with melatonin improves the circadian rhythms as well as autistic-like behavior [36], whereas daytime treatment was ineffective. The timed treatment of melatonin supplements or melatonin receptor agonists may prove to be an appropriate countermeasure. It will be important to examine whether this class of drugs may also be effective in reducing the epileptic discharges. Here it is worth noting that the epilepsy associated with focal cortical dysplasia and *Cntnap2* mutations is commonly resistant to pharmacological management and new treatments are urgently needed.

### Limitations

There are several limitations to this work that will need to be addressed in future studies. This study exclusively uses *Cntnap2* KO mice while ASD and epilepsy are genetically heterogeneous, and findings from one model may not generalize across other genetic backgrounds. Future research should assess whether the effects observed here are consistent across other ASD-related mouse models following DLaN exposure.

Additionally, while our findings showed that DLaN increases abnormal EEG activity, the link to seizures as well as the precise mechanism of action remains unclear. Specifically, it is not clear whether the effects of DLaN are due to circadian rhythm disruption, direct light exposure, or a combination. To address this, future studies should manipulate the circadian system independently of DLaN exposure to determine whether circadian misalignment alone is sufficient to drive the observed effects. Another limitation is that the estrous cycle of female mice was not tracked in this study. Given that fluctuations in ovarian hormones can significantly affect sleep architecture, this omission complicates the interpretation of sex differences. Future work should incorporate estrous cycle monitoring to more accurately assess sex-specific responses to DLaN exposure.

## Conclusion

Our study highlights the significant adverse effects of DLaN on sleep and neurological health, underscoring the importance of addressing this increasingly common environmental factor. Unlike many previous studies focusing on male rodents, we included both sexes and found that female mice were less vulnerable to DLaN-induced waking disruptions compared to males. *Cntnap2* KO were particularly sensitive to DLaN, showing more significant sleep disruptions and a sex-dependent effect on sleep phenotype.

A striking finding was the increased occurrence of abnormal EEG events under prolonged DLaN exposure. While *Cntnap2* KO mice naturally displayed abnormal EEG event due to their genetic deficit, we observed an increase in the occurrence under DLaN conditions, not only in *Cntnap2* KO mice, but also in the WT mice. This suggests that chronic DLaN exposure can alter brain networks, potentially exacerbating neurological vulnerabilities. Our results emphasize the urgent need to optimize lighting environments, particularly for individuals with ASD and epilepsy, who may be more sensitive to the effects of artificial light at night. Promoting sleep hygiene, such as minimizing exposure to dim light before and during sleep, could mitigate these risks. Ultimately, these findings not only advance our understanding of environmental factors like DLaN impact on vulnerable populations but also serve as a call to action to improve lighting practices for neurological and overall health.

## Methods

### Animals

13–15 weeks old 16 WT (8 female, 8 male) and KO mice (8 female, 10 male) were used in this study. All animal procedures were performed in accordance with the UCLA animal care committee’s regulations. *Cntnap2*^tm2Pele^ mutant mice backcrossed to the C57BL/6J background strain were acquired (B6.129(Cg)-*Cntnap2*^tm2Pele^/J, https://www.jax.org/strain/017482 [29]. *Cntnap2* null mutant (KO) and C57BL/6J wild-type (WT) mice were obtained from heterozygous breeding pairs. Weaned mice were genotyped (TransnetYX, Cordova, TN) and group-housed prior to experimentation. Mice were housed in light-tight ventilated cabinets in temperature- and humidity-controlled conditions, with free access to food and water.

### EEG/EMG surgery

At the age of 13–15 weeks, animals were anesthetized using ketamine/xylazine (100/8.8 mg/kg, intraperitoneal injection) and EEG/ EMG electrodes were implanted as described previously [70]. A prefabricated head mount (CAT:8201, Pinnacle Technologies, KS) was used to position four stainless-steel epidural screw electrodes. One front electrode was placed 1.5 mm anterior to bregma and 1.5 mm lateral to the central line, whereas the second two electrodes (interparietal—located over the visual cortex and common reference) were placed 2.5 mm posterior to bregma and 1.5 mm on either side. A fourth screw served as a ground. Electrical continuity between the screw electrode and head mount was aided by silver epoxy. EMG activity was monitored using stainless-steel Teflon-coated wires that were inserted bilaterally into the nuchal muscle. The head mount (integrated 2 × 3 pin grid array) was secured to the skull with dental cement. Mice were allowed to recover for at least 7 days before sleep recordings began.

### Experimental design

*Cntnap2* KO littermate and age-matched WT littermate controls were first entrained to a normal lighting cycle: 12 h light: 12 h dark (LD). Light intensity during the day was 300 lx as measured at the base of the cage, and 0 lx during the night. One week after surgery, mice were connected to a lightweight tether attached to a low-resistance commutator mounted over the cage (Pinnacle Technologies, KS) as previously described [70]. Mice were allowed a minimum of 2 additional days to acclimate to the tether and recording chamber. Subsequently, a baseline (BL) day was recorded, starting at lights on zeitgeber time 0. After the baseline recording, mice were housed under dim light at night (DLaN; ZT0-12: 300 lx; ZT12-24: 5 lx) for 2 weeks as described previously [36]. Two weeks later, the mice were attached to the commutator for the post 2 weeks DLaN recording for 24 h. After the recording, the mice were continuously housed under DLaN for another 4 weeks. After a total 6 weeks of DLaN, the mice were again attached to the commutator for the post 6 weeks DLaN recording for 24 h.

### EEG data acquisition and EEG power spectrum analysis

Data acquisition was done by Sirenia Acquisition software (Pinnacle Technologies, KS) as previously described [70]. EEG signals were low pass filtered with a 40 Hz cutoff and collected continuously at a sampling rate of 400 Hz. All data were recorded simultaneously in 1s epoch. Wake, NREM, and REM sleep were determined visually. Wake was scored when there was a high-EMG and low-EEG amplitude and high theta activity (EEG power density in the theta band, 6.0–9.0 Hz), concomitant with irregular, high EMG values. NREM sleep was sacored when there was a low-EMG and higher EEG amplitude compared with wake and high SWA. REM sleep was scored when the EMG and EEG amplitude was low, and high theta activity was visible in the EEG. Movement artifacts and abnormal EEG events were excluded for power spectral and slow-wave analysis. For the spectral analysis, complete and clean recordings are needed to enter the analysis. However, this was not possible in six animals (four male KO and two male WT mice), which were therefore excluded from the analysis of the absolute EEG power density spectra and SWA in NREM sleep. Spectral analysis was performed using fast Fourier transform (FFT; 0.1–40 Hz, 0.1 Hz resolution); the absolute power density spectra of waking and REM sleep in baseline and post DLaN days were analyzed.

### Data analysis

Three vigilance states (wake, NREM, and REM sleep) were scored offline in 10-s epochs by the Sirenia program. The manual scoring of vigilance states based on the EEG and EMG recordings was performed according to standardized criteria for mice [46]. The average amount of the vigilance states (wake, NREM sleep, and REM sleep) and EEG spectral data and NREM sleep SWA were analyzed in 1 h, 12 h and 24 h intervals.

Episode duration averages were compiled by the Sirenia program, which uses a conservative algorithm for bout lengths requiring sustained changes in arousal state to switch state. A bout was defined as 3 + continuous epochs, and the occurrence of 3 + continuous epochs of the different stage indicated the end of the current bout. Episode duration and bout number were analyzed in 12 h intervals.

To investigate the effect of DLaN on EEG power density of NREM sleep, we analyzed the relative EEG power density in the slow-wave range (SWA, 0.5–4.0 Hz) in NREM sleep as described previously [46]. Since for the slow wave activity analysis, it was necessary to re-calculate power density values relative to the control condition (WT, *n* = 13; KO, *n* = 16), complete and clean recordings were needed from all animals for both conditions to enter the analysis. Unfortunately, this was not possible in 3 animals under 2 weeks of DLaN and 4 of 6 weeks DLaN, which were therefore excluded from the analysis of SWA (2 weeks DLAN: WT, *n* = 11, KO, *n* = 15; 6 weeks DLaN: WT, *n* = 12, KO, *n* = 13).

### Abnormal EEG events identification

During vigilance state identification, occasional and distinct events of high amplitude EEG bursts were observed. Since alteration in E/I balance has been postulated as a mechanism underlying epileptogenesis and seizure generation, this abnormal EEG activity might be indicative of hypersynchronisation and/or epileptiform activity. Thus, these abnormal EEG events were marked on EEG traces (and excluded from the spectral analysis of the vigilance states described above). More precisely, the number and duration of abnormal events were quantified by marking them according to the following three criteria under either waking or REM sleep [41]: amplitude at least twice that of the background EEG signal, duration of at least one second and not caused by movement of the animal (no movement artifacts). Two events separated by less than 0.5 s were considered as a single one.

### Abnormal EEG events occurrence and power spectrum analysis

To evaluate this abnormal EEG events, we quantified their occurrence in 1-hr and 24-hr intervals. Given the differences in the amount of waking and REM sleep across the 24-hr cycle, we further normalized the frequency of these EEG events, calculating them as events per minute of REM sleep or waking under baseline conditions, after 2 weeks, and after 6 weeks of DLaN.

To better capture these EEG events, we score the EEG data in 1 s epoch during both normal REM sleep or waking states and during periods exhibiting abnormal EEG events. For each mouse, EEG power density was calculated in the 1–40 Hz range with a 1-Hz resolution for at least 10 abnormal events. These values were then compared to the power density recorded during normal waking or REM sleep occurring immediately before or after the abnormal EEG events.

### Statistical analysis

For data analysis, GraphPad Prism 10 (GraphPad software) was used. Anderson-Darling test and Shapiro-Wilk test (when *n* < 8) were used for the normality test before t-test. Unpaired student’s t-tests were used when distributions were normal; otherwise, nonparametric Mann-Whitney tests were used to determine statistically significant differences between groups. Paired student’s t-tests were used when distributions were normal; otherwise, the nonparametric Wilcoxon matched-pairs signed rank test was used to compare the difference between baseline and DLaN conditions. Unless noted otherwise, all other data were analyzed by mixed-effects model (REML) or two-way repeated measures ANOVA with the Geisser-Greenhouse correction. Bonferroni post hoc analyses followed any significant ANOVA tests. Mixed-effects models with Geisser–Greenhouse correction were applied to SWA and normalized REM event analyses to account for missing values. Detailed statistics results with samples size for each panel can be found in Supplemental Table 2.

## Supplementary Information

Below is the link to the electronic supplementary material.

Supplementary Material 1

Supplementary Material 2

## Data Availability

The datasets here used and analyzed are available from the corresponding author upon request.

## Abbreviations

Ank3-1b: Ank3-exon1b
ANOVA: Analysis of variance
ASD: Autism spectrum disorder
BL: Baseline
CDFE: Cortical dysplasia-focal epilepsy
Cntnap2: Contactin associated protein-like 2
DLaN: Dim light at night
EEG: Electroencephalograph
EMG: Electromyographic
E/I: Excitatory/inhibitory
Fmr1: Fragile X mental retardation syndrome 1
Nlgn: Neuroligin
NREM: Non rapid eye movement
n.s.: Not significant
PV +: Parvalbumin-positive
KO: Knockout
LD: Light-dark
SWA: Slow-wave activity
WT: Wild type
ZT: ZEITGEBER time
