# Supplementary material for: Dim light at night disrupts the sleep-wake cycle and exacerbates abnormal EEG activity in Cntnap2 knockout mice: implications for autism spectrum disorders
Source: Mol Autism. 2025 Dec 18;16:62. doi: 10.1186/s13229-025-00689-7 (PMC12713241; doi:10.1186/s13229-025-00689-7)
Supplement: Supplementary file 1 — Supplementary Material 1 [file 13229_2025_689_MOESM1_ESM.docx]

**Additional File 1
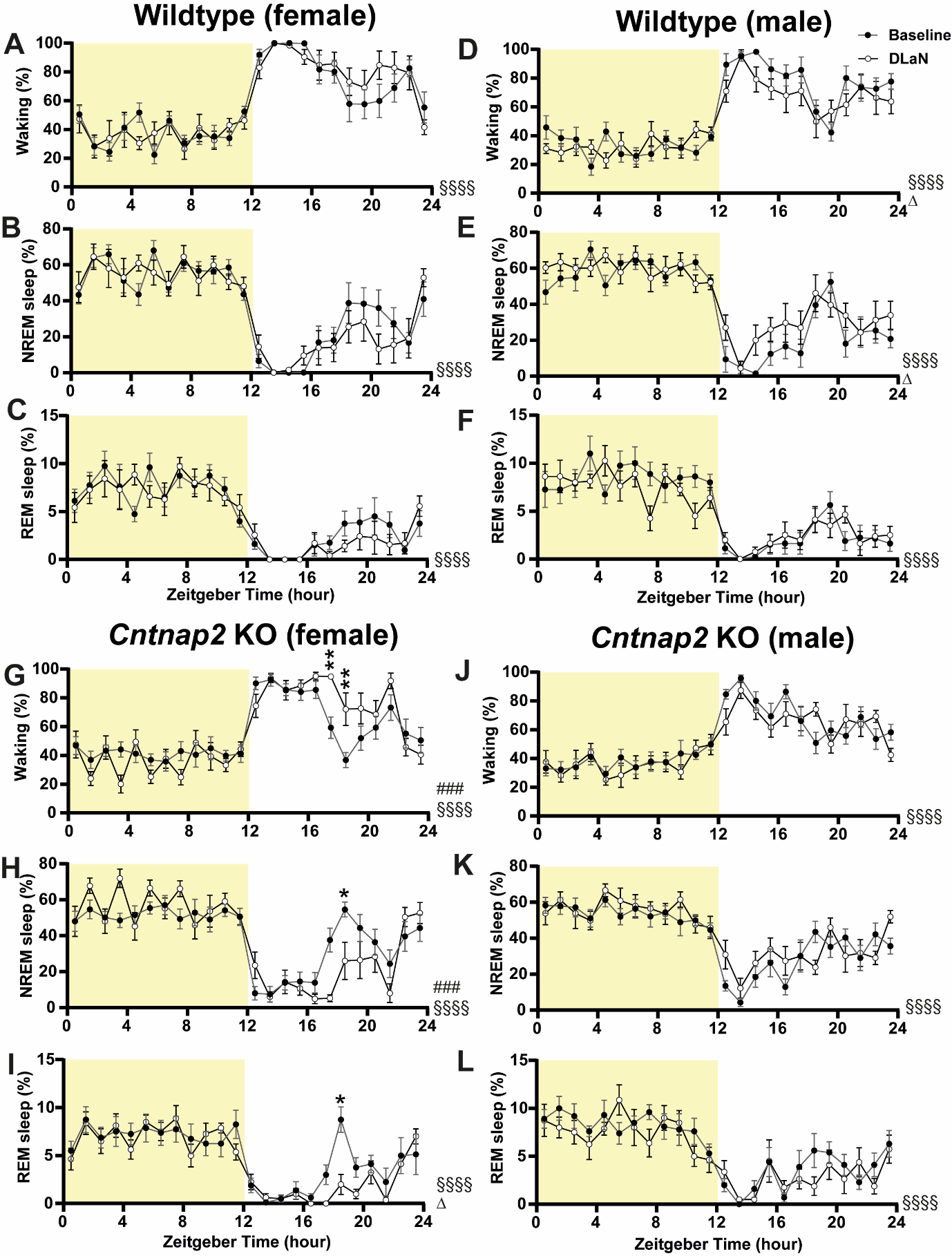
**

**Supplemental Figure 1. Sleep architecture of WT and *Cntnap2* KO mice during baseline and 2 weeks of DLaN.**

**A-L.** Twenty-four-hour distributions of time spent in wake, NREM sleep, and REM sleep for WT females (**A-C**), WT males (**D-F**), KO females (**G-I**) and KO males (**K-M**) during baseline (black circle) and after 2 weeks of DLaN (white circle). Pound sign (#) indicates a significant interaction between the factors “zeitgeber time” and “DLaN” (two-way repeated-measures ANOVA with Geisser-Greenhouse's correction; ###p<0.001). Main effect of “zeitgeber time” and “DLaN” were showed as “§” and “∆” (§§§§p < 0.0001, ∆p<0.05). Asterisks indicate significant post-hoc differences between baseline and 2 weeks of DLaN (Bonferroni multiple comparisons test, *p < 0.05, **p<0.01). Sample size: WT female, baseline=8 and DLaN=7; WT male, baseline=8 and DLaN=8; KO female, baseline=8 and DLaN=8; KO male, baseline=10 and DLaN=8. Data are shown as mean ±SEM.


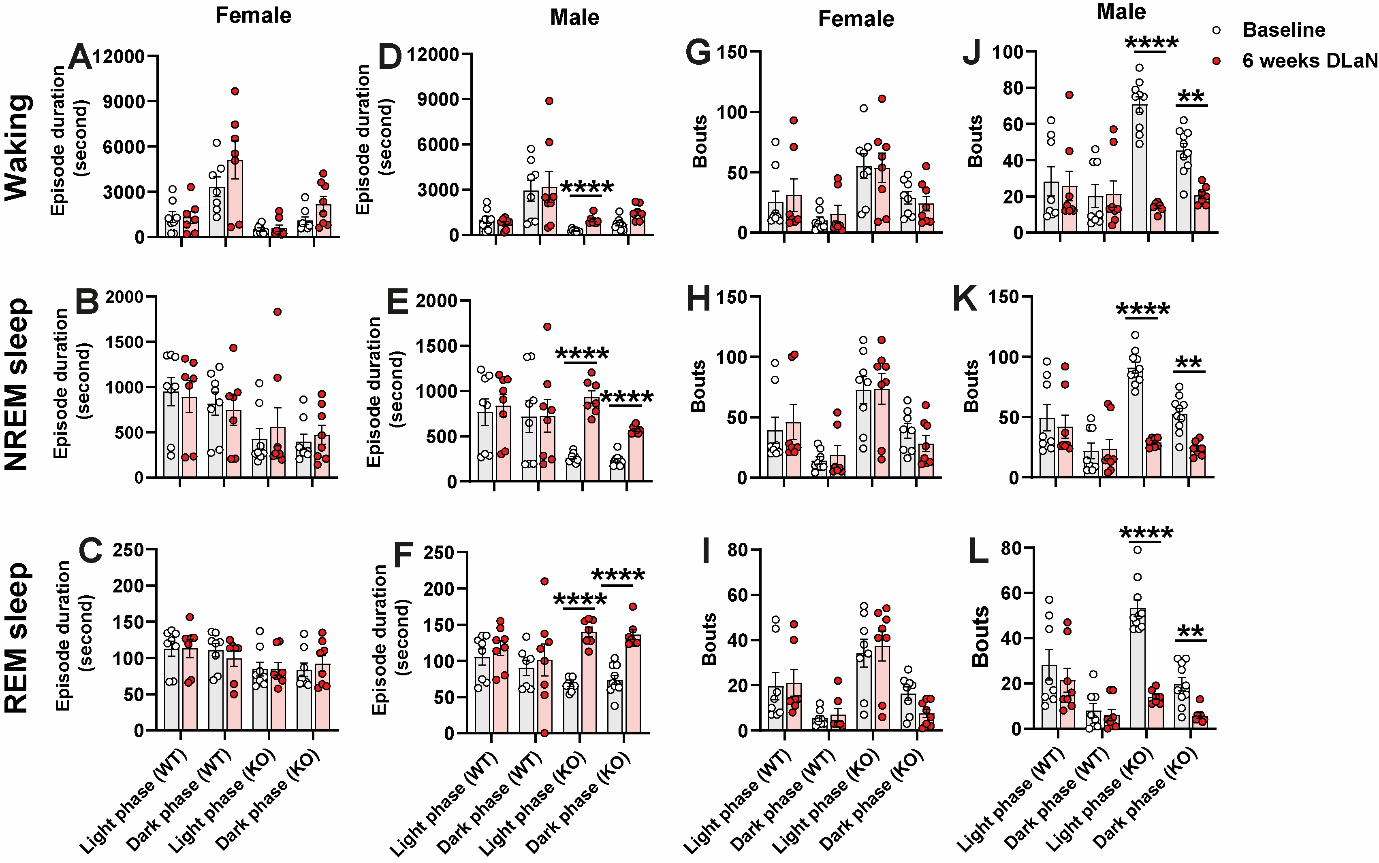


**Supplemental Figure 2. Episode duration and bout number in baseline and DLaN in WT and *Cntnap2* KO mice.**

**A–F.** Episode duration of wake, NREM sleep, and REM sleep for baseline (white circle) and 6 weeks DLaN (red circle) in female (**A, B, C**) and male (D, E, F) mice. G-L. Bout number of waking, NREM sleep, and REM sleep for baseline (white circle) and 6 weeks DLaN (red circle) in female (**A, B, C**) and male (**D, E, F**) mice. Asterisks indicate significant differences between baseline and DLaN (*p < 0.05, **p < 0.01, ***p < 0.001, ****p < 0.0001, paired t -test or Wilcoxon matched-pairs signed rank test). Sample size: WT female, baseline=8 and DLaN=7; WT male, baseline=8 and DLaN=8; KO female, baseline=8 and DLaN=8; KO male, baseline=10 and DLaN=7. Data are shown as mean ±SEM.

**
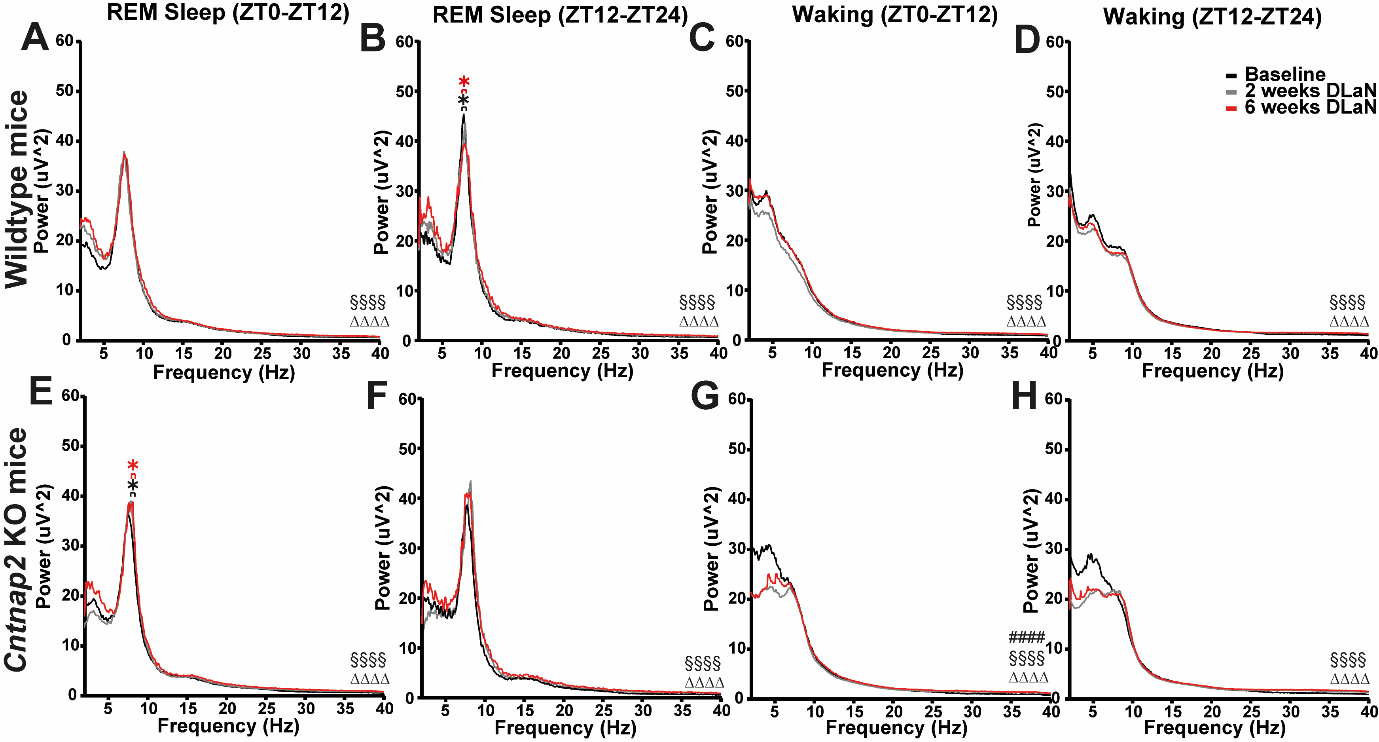
Supplemental Figure 3. Effect of DLaN on the power spectrum of REM sleep and waking.**

**A-H.** Absolute EEG power spectrum during REM sleep in the light phase (**A, E**) and dark/dim light phase (**B, F**) for baseline (black), 2 weeks of DLaN (gray), and 6 weeks of DLaN (red) in WT mice and KO mice. Absolute EEG power spectrum during wake in the light phase (**C, G**) and dark/dim light phase (**D, H**) for baseline (black), 2 weeks of DLaN (gray), and 6 weeks of DLaN (red) in WT and KO mice. Pound sign (#) indicates a significant interaction between the factors “Frequency” and “DLaN” (two-way ANOVA; ####p<0.0001). Main effect from two-way ANOVA of “Frequency” and “DLaN” were showed as “§” and “∆” (§§§§p < 0.0001, ∆∆∆∆p<0.0001). Asterisks indicate significant differences between baseline and DLaN (p = 0.05–0.0001, Bonferroni multiple comparisons test). The frequency bin size is 0.1 Hz and X axis started from 3 Hz. n = 11–16 mice per genotype and condition. Data are shown as the mean.


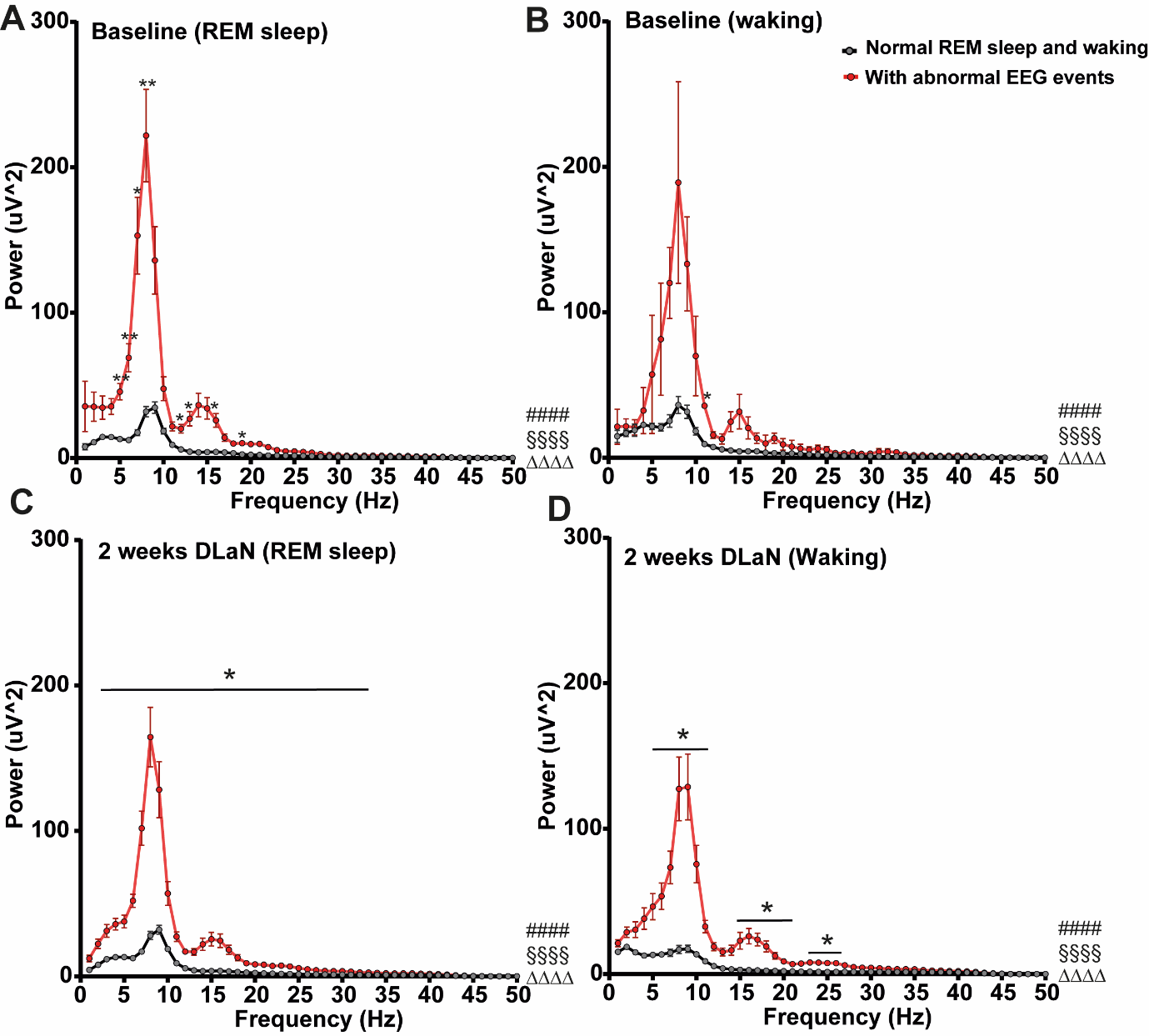


**Supplemental Figure 4. Abnormal EEG power analysis under baseline and 2 weeks of DLaN**

**A-D.** Absolute EEG power spectrum during REM sleep (**A, C**) and waking (**B, D**) with abnormal EEG event occurrence (red) and without abnormal EEG event occurrence (black) for baseline and 2 weeks DLaN in *Cntnap2* KO mice. Pound sign (#) indicates a significant interaction between the factors “Frequency” and “abnormal EEG event” (two-way repeated measures ANOVA; ####p<0.0001). Main effect from two-way ANOVA of “Frequency” and “abnormal EEG event” were showed as “§” and “∆” (§§§§p < 0.0001, ∆∆∆∆p<0.0001). Asterisks indicate significant differences between normal and abnormal EEG events (p = 0.05–0.0001, Bonferroni multiple comparisons test). The frequency bin size is 1 Hz. n = 12–16 mice in panel A, C, D, n = 5 in panel B. Data are shown as mean ± SEM.
